# Genetic variation associated with childhood and adult stature and risk of *MYCN*‐amplified neuroblastoma

**DOI:** 10.1002/cam4.3458

**Published:** 2020-09-17

**Authors:** Eleanor C. Semmes, Erica Shen, Jennifer L. Cohen, Chenan Zhang, Qingyi Wei, Jillian H. Hurst, Kyle M. Walsh

**Affiliations:** ^1^ Medical Scientist Training Program Duke University Durham NC USA; ^2^ Department of Pediatrics Children's Health and Discovery Institute Duke University Durham NC USA; ^3^ Division of Neuro‐epidemiology Department of Neurosurgery Duke University Durham NC USA; ^4^ Division of Medical Genetics Department of Pediatrics Duke University Durham NC USA; ^5^ Department of Epidemiology and Biostatistics University of California San Francisco CA USA; ^6^ Department of Population Health Sciences Duke University School of Medicine Durham NC USA; ^7^ Duke Cancer Institute Duke University Medical Center Durham NC USA

**Keywords:** cancer genetics, Cancer Medicine, cancer risk factors, epidemiology, genome‐wide association, height, MYCN, neuroblastoma, pathway analysis, pediatric cancer, polygenic score, stature

## Abstract

**Background:**

Neuroblastoma is the most common pediatric solid tumor. *MYCN*‐amplification is an important negative prognostic indicator and inherited genetic contributions to risk are incompletely understood. Genetic determinants of stature increase risk of several adult and childhood cancers, but have not been studied in neuroblastoma despite elevated neuroblastoma incidence in children with congenital overgrowth syndromes.

**Methods:**

We investigated the association between genetic determinants of height and neuroblastoma risk in 1538 neuroblastoma cases, stratified by *MYCN*‐amplification status, and compared to 3390 European‐ancestry controls using polygenic scores for birth length (five variants), childhood height (six variants), and adult height (413 variants). We further examined the UK Biobank to evaluate the association of known neuroblastoma risk loci and stature.

**Results:**

An increase in the polygenic score for childhood stature, corresponding to a ~0.5 cm increase in pre‐pubertal height, was associated with greater risk of *MYCN*‐amplified neuroblastoma (OR = 1.14, *P* = .047). An increase in the polygenic score for adult stature, corresponding to a ~1.7 cm increase in adult height attainment, was associated with decreased risk of *MYCN*‐amplified neuroblastoma (OR = 0.87, *P* = .047). These associations persisted in case‐case analyses comparing *MYCN*‐amplified to *MYCN*‐unamplified neuroblastoma. No polygenic height scores were associated with *MYCN*‐unamplified neuroblastoma risk. Previously identified genome‐wide association study hits for neuroblastoma (N = 10) were significantly enriched for association with both childhood (*P* = 4.0 × 10^−3^) and adult height (*P* = 8.9 × 10^−3^) in >250 000 UK Biobank study participants.

**Conclusions:**

Genetic propensity to taller childhood height and shorter adult height were associated with *MYCN*‐amplified neuroblastoma risk, suggesting that biological pathways affecting growth trajectories and pubertal timing may contribute to *MYCN*‐amplified neuroblastoma etiology.

## Graphical abstract

We investigated the association between genetic determinants of height and neuroblastoma risk in 1538 neuroblastoma cases, stratified by *MYCN*‐amplification status, and compared to 3390 European‐ancestry controls using polygenic scores for birth length, childhood height, and adult height. Genetic propensity to taller childhood height and shorter adult height were associated with *MYCN*‐amplified neuroblastoma risk, suggesting that biological pathways affecting growth trajectories and pubertal timing may contribute to *MYCN*‐amplified neuroblastoma etiology. Graphical abstract created with BioRender.com and height icon by Becris from the Noun Project.

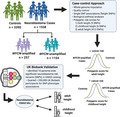

## 1 INTRODUCTION

Neuroblastoma is the most common solid tumor of childhood,^1^ accounting for 8%‐10% of all pediatric cancers and responsible for up to 15% of cancer‐related childhood mortality.^2^, ^3^, ^4^ Clinical patterns range across a wide spectrum, from spontaneous regression to poorly differentiated and aggressive phenotypes.^5^ One of the most well‐known prognostic indicators for neuroblastoma is genomic amplification of *MYCN* (Homo sapiens v‐myc myelocytomatosis viral‐related oncogene, neuroblastoma derived), present in ~20% of patients. *MYCN‐*amplification is consistently associated with an aggressive neuroblastoma phenotype and poorer clinical outcomes.^2^, ^3^, ^6^, ^7^ *MYCN* is a member of the MYC family of transcription factors, important in survival, proliferation, and differentiation processes related to tumor initiation and progression.^8^, ^9^

The genetic etiology of neuroblastoma has increasingly been revealed by genome‐wide association studies (GWAS), which indicate that sporadic neuroblastoma is a polygenic disease with ten independent susceptibility loci identified to‐date.^10^, ^11^, ^12^, ^13^, ^14^ Several alleles appear to specifically confer risk of *MYCN*‐amplified neuroblastoma, indicating that genetic risk variants may be specific to certain molecular subtypes of neuroblastoma.^15^, ^16^, ^17^ The causative variants and biologic mechanisms linking these risk loci to neuroblastoma pathogenesis, and *MYCN*‐amplified tumorigenesis specifically, remain an active area of investigation.

Epidemiologic studies have observed strong relationships between height attainment and risk of various adult malignancies (eg, breast, colorectal, thyroid, ovarian, endometrial, and prostate), and these associations have been supported by recent Mendelian randomization analyses.^18^, ^19^, ^20^, ^21^, ^22^, ^23^, ^24^ A recent case‐control study that leveraged polygenic scores as instrumental variables observed significant associations between genetic determinants of childhood and adult height attainment and risk of childhood osteosarcoma.^25^ Associations between genetic determinants of stature and risk of other childhood cancers, including neuroblastoma, have not been evaluated.

It is possible that biological pathways underlying childhood growth and adult height attainment overlap those involved in neuroblastoma etiology. This is supported by a number of congenital overgrowth syndromes that are associated with increased risk for neuroblastoma (eg, Beckwith‐Wiedemann, Costello, Simpson‐Golabi‐Behmel, Sotos, and Weaver).^26^, ^27^, ^28^, ^29^, ^30^ To test this hypothesis, we investigated the association of genetic determinants of stature and risk of neuroblastoma, stratifying analyses by *MYCN*‐amplification status given previous studies identifying non‐overlapping genetic risk variants underlying *MYCN*‐amplified vs *MYCN*‐unamplified neuroblastoma risk. The association of previously published and externally validated polygenic scores for birth length, pre‐pubertal height attainment, and adult height attainment with risk of neuroblastoma was evaluated in 1538 cases and 3390 controls of European ancestry. We also explored whether neuroblastoma risk loci identified in prior GWAS were enriched for association with childhood and adult stature among UK Biobank participants to further explore connections between genetic determinants of stature and subtype‐specific neuroblastoma risk.

## 2 MATERIALS AND METHODS

### 2.1 Neuroblastoma cases and controls

A total of 1538 neuroblastoma cases and 3390 controls of European ancestry were included. Neuroblastoma cases were non‐Hispanic white patients <19 years old at diagnosis who were recruited by the Children's Oncology Group and Children's Hospital of Philadelphia, as described previously.^11^ Genotyping data for cases were downloaded from dbGaP study accession phs000124.v2.p1, whereas genotyping data for the controls was retrieved from Illumina's iControlsDB database. Samples from both cases and controls were genotyped on the Illumina HumanHap550 genome‐wide single nucleotide polymorphism (SNP) array, a standard commercially available platform.

Genotyping quality control measures were implemented for both cases and controls as described previously.^31^ Briefly, SNPs with genotyping call rates <98%, SNPs with Hardy‐Weinberg equilibrium *P* < .0001 among controls, subjects with genotyping call rates <97%, those with non‐European ancestry as determined from principal components analysis (ie, subjects that fell more than three SDs from mean CEU values), evidence of cryptic relatedness (defined as identity by descent proportion >0.20) and those with discrepant sex between genotyping data and clinical report were removed from analyses.

### 2.2 Genotype imputation

Genome‐wide association study data underwent genome‐wide imputation as previously described.^25^ Haplotype phasing was performed with SHAPEIT (version 2.790), and whole‐genome imputation was performed using Minimac3 with 64 976 human haplotypes from the 2016 Haplotype Reference Consortium used as the imputation reference panel.^32^, ^33^, ^34^ SNPs with imputation quality (info) scores <0.60 or posterior probabilities <.90 were excluded.^35^

### 2.3 Polygenic score construction and single SNP analyses

We constructed polygenic scores for birth length, childhood height attainment, and adult height attainment using previously published genome‐wide significant variants.^36^, ^37^, ^38^, ^39^ Childhood height was assessed prior to adult height attainment and to the standard peak of pubertal growth acceleration, measured at age 10 in girls and age 12 in boys. Weighted polygenic scores for case and control subjects were generated using PLINK,^40^ with the effect of individual SNPs weighted according to the beta value and direction of effect from prior GWAS. For the height scores, we used five variants for birth length, six variants for childhood height, and 413 variants for adult height attainment after selecting the most statistically significant SNP from each independent stature‐associated locus (Table S1).^25^ We also created a reduced polygenic height score for adult height attainment that included only 407 variants, excluding six SNPs in linkage disequilibrium (LD) with those contained in the childhood height score.^37^, ^38^ These polygenic scores were previously applied in other published studies and have shown strong correlation with stature.^25^ We used US national height statistics to standardize the polygenic height scores in terms of centimeters of predicted height, as previously published.^25^, ^41^ A 1 SD change in the polygenic scores correspond to approximately 0.14 cm in birth length, 0.5 cm in childhood height, and 1.7 cm in adult height.^36^

We performed logistic regression analyses to test for association between the polygenic scores and neuroblastoma risk, adjusted for five ancestry‐informative principal components and stratified by *MYCN*‐amplification status (*MYCN*‐amplified N = 257, *MYCN*‐unamplified N = 1154, unknown *MYCN* status N = 127). We also examined all SNPs for single‐SNP associations with neuroblastoma case‐control status and *MYCN*‐amplified neuroblastoma case‐control status using logistic regression, adjusting for five principal components. In single‐SNP association analyses, we included one additional variant previously associated with late pubertal growth (rs7759938).^37^

### 2.4 Pathway analysis of height‐associated SNPs

For each of the 407 SNPs comprising our reduced polygenic score for adult height attainment, we identified the nearest protein‐coding gene in the GRCh37 assembly.^25^, ^38^ RefSeq gene names were queried in two pathway‐analysis databases: the Kyoto Encyclopedia of Genes and Genomes (KEGG) and the Protein ANalysis THrough Evolutionary Relationships (PANTHER) Classification System. After identifying highly represented biological pathways within the height‐associated gene list (KEGG *P* ≤ .001; PANTHER gene count ≥ 10), we recalculated the polygenic height scores, this time limiting the number of SNPs contributing to the model to only those residing within each respective pathway. In this manner, we sought to determine whether any specific biological processes contributing to height attainment were driving the association between the polygenic score and subtype‐specific neuroblastoma risk (Table S2).

### 2.5 Identification of neuroblastoma risk loci from prior GWAS

We accessed the NHGRI‐EBI Catalog of genome‐wide association studies (GWAS catalog; https://www.ebi.ac.uk/gwas/) to compile a curated list of GWAS loci associated with neuroblastoma risk at genome‐wide statistical significance (*P* < 5.0 × 10^−8^).^42^ We pruned the list of significant variants for LD (*R* ^2^ ≤ .15 in European‐ancestry populations) using the NCI LDlink tool and cross‐referenced this list with published reviews on neuroblastoma GWAS.^43^

### 2.6 Control SNP sets

In addition to the set of neuroblastoma risk SNPs, we also created comparison SNP sets to serve as negative controls to test for enrichment of stature‐associated SNPs among neuroblastoma GWAS hits. A set of unlinked control SNPs from the 1000 Genomes Project reference panel was generated using SNPsnap (Broad Institute).^44^ Control variants were matched to the 10 neuroblastoma risk SNPs on minor allele frequency (±5%), surrounding gene density (±50%), distance to nearest gene (±50%) and, as a proxy for haplotype block size, the number of other SNPs in LD at *R* ^2^ ≥ .50 (±50%). Four control SNPs were identified for each input SNP, resulting in a total set of 40 SNPsnap control SNPs (Table S3).

Because neuroblastoma risk SNPs are trait‐associated variants that may be likelier to associate with additional traits, we also identified a second set of control SNPs by querying the GWAS catalog for glioma/glioblastoma SNPs using the same methodology as for neuroblastoma. This yielded 19 unlinked glioma/glioblastoma‐associated variants to be used as a second control SNP set (Table S3).

### 2.7 *eQTL and* in silico *SNP functional analyses*

We further characterized neuroblastoma risk SNPs and control SNPs using the HaploReg database, which annotates chromatin state and regulatory motifs of specific variants and LD‐block regions surrounding a given SNP.^45^ We examined whether variants were expression quantitative trait loci (eQTLs), protein‐binding, located in DNAse hypersensitive sites, promoter or enhancer histone marks, or were predicted to change transcription factor binding motifs.

### 2.8 UK Biobank GeneATLAS analyses

The atlas of genetic associations from the UK Biobank (GeneATLAS; http://geneatlas.roslin.ed.au.uk/) was constructed by genotyping ~450 000 European‐ancestry individuals for 805 426 genetic variants, performing genome‐wide SNP imputation and quality‐control, then linking genetic data to electronic health record data.47 GeneATLAS contains data for 778 traits (118 quantitative, 660 binary) and their associations with 9 113 133 genetic variants (genotyped and imputed after quality control) across 452 264 individuals. GeneATLAS is a searchable database that can be queried for genetic data (eg, variant, gene, chromosome region) or phenotypic data (eg, traits such as height).^46^

We queried the GeneATLAS database for the 10 genetic variants previously associated with risk of neuroblastoma as well as our two control SNP sets to identify any associations of these SNPs with “adult standing height” and “comparative height size at age 10.” We then assessed how many neuroblastoma risk loci were associated with stature (at *P* < .01) and compared these loci to results for the two control SNP sets to determine if neuroblastoma risk SNPs were more likely to be associated with stature than control SNPs, using Fisher's exact tests.

## 3 RESULTS

**Figure 1:**
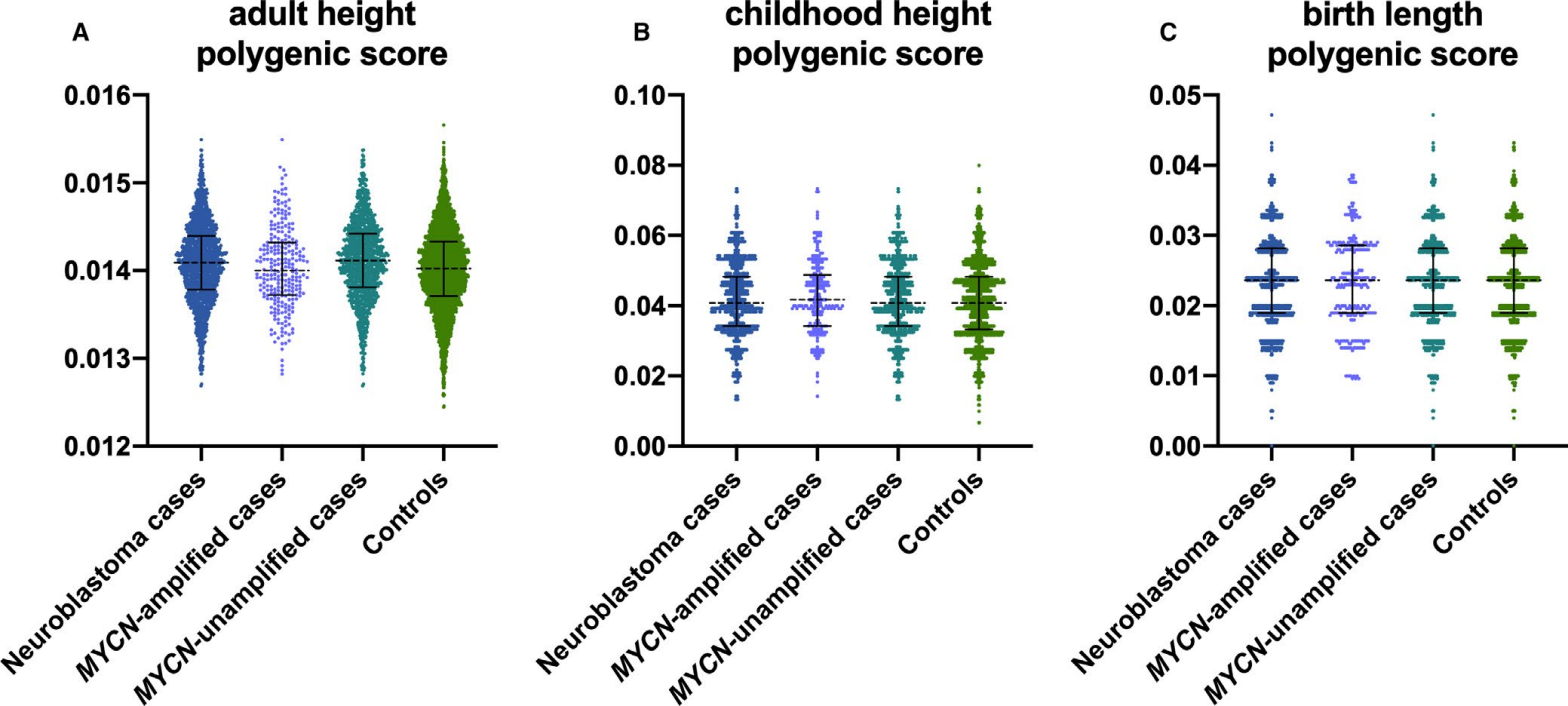
Distribution of polygenic height scores in neuroblastoma patients and controls. The distribution of the polygenic scores for (A) adult height, (B) childhood height and (C) birth length were compared across all neuroblastoma patients (N = 1538), *MYCN*‐amplified patients (N = 257), *MYCN*‐unamplified patients (N = 1154), and controls (N = 3390). Black bars indicate median and interquartile range

Polygenic scores were constructed using previously published genome‐wide significant variants for birth length (N = 5 SNPs), childhood height (N = 6 SNPs), and adult height (N = 413 SNPs) and examined for association in a case‐control set of 1538 neuroblastoma cases (257 *MYCN*‐amplified, 1154 *MYCN*‐unamplified) and 3390 controls of European ancestry with adjustment for five ancestry‐informative principal components. The polygenic score for birth length was not associated with neuroblastoma risk overall (*P* = .78) or *MYCN*‐amplified neuroblastoma risk (*P* = .39) in case‐control analyses (Table 1). Childhood and adult height attainment polygenic scores were also not associated with neuroblastoma risk overall (*P* = .68 and *P* = .55 respectively); however, both were associated with *MYCN*‐amplified neuroblastoma risk (Table 1). A 1 SD increase in the polygenic score for childhood height, corresponding to an approximately 0.5 cm increase in pre‐pubertal stature, was associated with greater risk of *MYCN*‐amplified neuroblastoma in case‐control analyses (OR = 1.14, *P* = .047) and was suggestively associated when *MYCN*‐amplified patients were compared to *MYCN*‐unamplified patients in case‐case analyses (OR = 1.13, *P* = .083). In contrast, a 1 SD increase in the polygenic score for adult height, corresponding to an approximately 1.7 cm increase in adult height attainment, was associated with decreased risk of *MYCN*‐amplified neuroblastoma in both case‐control (OR = 0.87, *P* = .047) and case‐case (*MYCN*‐amplified vs *MYCN*‐unamplified) analyses (OR = 0.85, *P* = .028; Table 1). While the median polygenic height scores differed across neuroblastoma patients and controls, the overall shapes of the distributions were very similar (Figure 1). We did not observe any case‐control associations when polygenic score analyses were stratified by age of onset (<18 months, 18 months‐12 years, 12‐19 years), histologic grade, or ploidy, after controlling for *MYCN* status.

**Table 1:** Multivariate logistic regression analyses for polygenic height scores and neuroblastoma risk in European‐ancestry subjects, overall and by *MYCN* status^a^

Polygenic score	Case‐control			MYCN‐amplified case‐control			MYCN‐amplified case‐case		
	OR^b^	95% CI	*P*‐value	OR^c^	95% CI	*P*‐value	OR^d^	95% CI	*P*‐value
Birth length	1.01	(0.94‐1.08)	.78	1.06	(0.93‐1.19)	.39	1.05	(0.92‐1.19)	.47
Childhood height	1.01	(0.95‐1.08)	.68	1.14	(1.00‐1.3)	**.047**	1.13	(0.98‐1.30)	.083
Adult height	0.98	(0.91‐1.05)	.55	0.87	(0.76‐1.0)	**.047**	0.85	(0.74‐0.98)	**.028**
Adult height (reduced)^e^	0.97	(0.90‐1.04)	.44	0.83	(0.69‐0.97)	**8.8 × 10^−3^**	0.82	(0.67‐0.96)	**5.7 × 10^−3^**
Adult height (reduced), and Childhood height^f^	0.97	(0.90‐1.04)	.44	0.83	(0.69‐0.97)	**8.9 × 10^−3^**	0.82	(0.68‐0.96)	**6.3 × 10** ^−^ **^3^**
	1.01	(0.95‐1.08)	.68	1.14	(1.01‐1.28)	**.048**	1.13	(0.99‐1.28)	.092

Note

*P*‐values < 0.05 in bold.

Abbreviations: 95% CI, 95% confidence interval; MYCN, homo sapiens v‐myc myelocytomatosis viral‐related oncogene, neuroblastoma derived; OR, odds ratio.

^a^ Multivariate logistic regression, adjusted for sex and top five ancestry‐informative principal components in all analyses.

^b^ OR represents the neuroblastoma risk associated with a 1 SD increase in the polygenic height score, which corresponds to approximately 0.14 cm in birth length, 0.5 cm in childhood height, and 1.7 cm in adult height.

^c^ OR represents the *MYCN*‐amplified neuroblastoma risk associated with a 1 SD increase in the polygenic height score, which corresponds to approximately 0.14 cm in birth length, 0.5 cm in childhood height, and 1.7 cm in adult height.

^d^ OR represents the risk of having MYCN‐amplification among neuroblastoma cases associated with a 1 SD increase in the polygenic height score, which corresponds to approximately 0.14 cm in birth length, 0.5 cm in childhood height, and 1.7 cm in adult height.

^e^ Polygenic score for adult height (reduced) excludes six SNPs in linkage disequilibrium with childhood height SNPs.

^f^ Jointly modeled polygenic scores for adult height (reduced) and childhood height, with adjustment for five ancestry‐informative principal components.

In a reduced model that removed 6 adult height SNPs in LD with childhood height SNPs, the association between the polygenic score for adult height and risk of *MYCN*‐amplified neuroblastoma was strengthened (OR_case‐control_ = 0.83, *P*_case‐control_ = 8.8 × 10^−3^; and OR_case‐case_ = 0.82; *P*_case‐case_ = 5.7 × 10^−3^). Results remained significant when including both the reduced polygenic score for adult height as well as the polygenic score for childhood height together in regression models (Table 1).

Single SNP analyses identified 28 SNPs that were nominally associated with neuroblastoma risk at *P* < .05, all of which were associated with adult height attainment (Table S4). No single height‐associated SNPs were significantly associated with neuroblastoma risk after Bonferroni correction for multiple comparisons. Additionally, 24 variants were nominally associated with *MYCN*‐amplified neuroblastoma risk at *P* < .05, 23 of which were associated with adult height attainment and 1 of which was associated with childhood height attainment (Table S5), yet none were significantly associated with *MYCN*‐amplified neuroblastoma risk after Bonferroni correction. Thus, individual genetic variants that influence stature appeared to contribute only modest risk of neuroblastoma or of the *MYCN*‐amplified subtype of neuroblastoma.

To investigate possible biological pathways underlying these associations, SNPs associated with adult height attainment were mapped to the nearest gene and the genes were mapped to associated biological pathways using the KEGG and PANTHER databases.^25^ Hedgehog signaling, WNT signaling, gonadotropin‐releasing hormone receptor signaling, cholecystokinin receptor signaling, inflammation, and integrin signaling pathways were enriched for height‐associated genes and used to create pathway‐specific polygenic scores. No pathway‐specific polygenic scores were associated with *MYCN*‐amplified neuroblastoma risk (Table S6). Due to the small number of SNPs available for constructing the birth length (N = 5) and childhood height (N = 6) polygenic scores, we could not complete pathway‐specific analyses for these variants.

We sought to validate our case‐control analyses suggestive of an association between genetic contributions to stature and *MYCN*‐amplified neuroblastoma risk using additional genotype‐phenotype datasets. We could not perform a formal replication analysis in an independent case‐control dataset due to the limited incidence of neuroblastoma, which greatly limits sample sizes for genomic studies in pediatric malignancies. Using the GWAS catalog, we identified 10 independent genome‐wide significant risk variants for neuroblastoma (Table 2) and two sets of comparison SNPs to serve as controls to explore whether previously identified GWAS risk loci for neuroblastoma were associated with height phenotypes in publicly available datasets. The two control sets included (a) 40 SNPs matched on minor allele frequency, gene density, distance to nearest gene, and haplotype structure, and (b) 19 SNPs previously associated with glioma/glioblastoma risk, an adult‐onset malignancy of nervous system tissue. Functional annotation and in silico analysis of the neuroblastoma SNP set and these two control SNP sets demonstrated similar characteristics in terms of impact on chromatin structure and gene expression (Table S3). We tested these SNPs for association with “adult standing height” and “comparative height size at age 10” among >450 000 patients from the UK Biobank database (Table 2). Notably, 6/10 neuroblastoma risk SNPs were associated with height at age 10 years compared to only 5/40 control SNPs (*P* = 4.0 × 10^−3^) and 2/19 glioma SNPs (*P* = 8.9 × 10^−3^), indicating that neuroblastoma risk loci are enriched for association with childhood height attainment. Additionally, 6/10 neuroblastoma risk SNPs were associated with adult height, compared to only 6/40 control SNPs (*P* = 7.3 × 10^−3^) and 3/19 glioma SNPs (*P* = .032), further highlighting the enrichment of neuroblastoma risk variants for associations with stature.

**Table 2:** Summary of genome‐wide significant neuroblastoma risk SNPs and associations with UK Biobank height phenotypes

Author (y)	Locus	Lead SNP	NB risk allele	Gene	*P*_Height at age 10 (childhood)_	*P*_Standing height (adulthood)_
Capasso et al (2009)	2q35	rs6435862	G	*BARD1*	.72	.12
McDaniel et al (2017)	3q25.32	rs6441201^a^	A	*RSRC1*	4.5 × 10^−4^	1.5 × 10^−22^
McDaniel et al (2017)	4p16.1	rs3796727^a^	A	*CPZ*	2.7 × 10^−3^	3.2 × 10^−5^
Diskin et al (2012)	6q16.3	rs72990858	A	*HACE1*	.059	.43
Diskin et al (2012)	6q16.3	rs17065417^b^	C	*LIN28B*	.72	7.4 × 10^−7^
Wang et al (2010)	6p22.3	rs4712653^b^	C	*CASC15*	3.4 × 10^−3^	2.3 × 10^−9^
Chang et al (2017)	11q22.2	rs10895322	G	*MMP20*	.33	.75
Diskin et al (2012)	11p11.2	rs11037575^b^	C	*HSD17B12*	4.9 × 10^−14^	.16029
McDaniel et al (2017)	11p15.4	rs2168101^b^	C	*LMO1*	8.8 × 10^−11^	3.5 × 10^−15^
Diskin et al (2012)	17p13.1	rs35850753^a^	T	*TP53*	5.1 × 10^−7^	1.7 × 10^−22^

Abbreviations: MYCN, homo sapiens v‐myc myelocytomatosis viral‐related oncogene, neuroblastoma derived; NB, neuroblastoma; SNPs, single‐nucleotide polymorphism.

^a^ Neuroblastoma risk allele associated with taller stature. Association data extracted from genome‐wide association study (GWAS) of >250 000 UK Biobank participants.

^b^ Neuroblastoma risk allele associated with shorter stature. Association data extracted from GWAS of >250 000 UK Biobank participants.

Although neuroblastoma risk loci were enriched for association with both childhood and adult height phenotypes among UK Biobank participants, not all of these variants were associated with the same direction of effect on height. Three neuroblastoma risk alleles were associated with taller adult height and three were associated with shorter adult height (Table 2). Similarly, divergent directions of effect were observed for childhood height, suggesting that while neuroblastoma risk loci may be significantly more likely to impact growth phenotypes, stature itself is unlikely to mediate the association between these GWAS risk alleles and neuroblastoma pathogenesis. Notably, the UK Biobank data provides additional support for our case‐control results, implicating genetic determinants of height in neuroblastoma etiology and a role for shared biological pathways influencing both height and neuroblastomagenesis.

## 4 DISCUSSION

Polygenic score analyses revealed that each 0.5 cm increase in genetically predicted childhood height attainment prior to puberty was associated with a 1.14‐fold increase in risk of *MYCN*‐amplified neuroblastoma. Conversely, each 1.7 cm increase in genetically predicted adult height attainment was associated with a 0.86‐fold decrease in *MYCN*‐amplified neuroblastoma risk. When considering the distribution of polygenic risk scores across and between groups, there was little evidence that the *MYCN*‐amplified neuroblastoma patients were enriched for extreme outlier polygenic scores that would skew analyses. Instead, the differences in patients and controls seemed to be due to general shifts in the polygenic scores in *MYCN‐*stratified analyses. When polygenic scores for childhood and adult height were modeled together, they were independently associated with neuroblastoma risk with effects in opposing directions. All logistic regression models were adjusted for sex and the top five ancestry‐informative principal components. Common biology underlying height attainment and neuroblastoma development has not previously been examined in the context of shared genetic architecture, despite a clinical association between overgrowth syndromes and neuroblastoma risk that has been known for decades; however, our results strongly suggest pleiotropy whereby common genes or biological pathways are important for both stature and neuroblastomagenesis.

Numerous congenital overgrowth syndromes are associated with neuroblastoma occurence,^47^ including: EZH2‐related Weaver syndrome (online mendelian inheritance in man, OMIM #277590), Sotos syndrome^48^ (OMIM #117550), Beckwith‐Wiedemann syndrome (BWS; OMIM # 130650), Simpson‐Golabi‐Behmel syndrome^49^ (OMIM #312870), and Costello syndrome^50^ (OMIM #218040). Some of these syndromes, such as BWS, have accompanying tumor screening guidelines. There are numerous other overgrowth syndromes, not listed here, that increase tumor risk more generally, but which have not shown an increased occurrence of neuroblastoma specifically.^47^ The tumor epidemiology in Sotos syndrome is different from other overgrowth syndromes in that embryonal tumors, while occurring, do not comprise the majority of tumors in this syndrome.^47^ In patients with BWS, neuroblastoma accounts for 5% of tumors.^47^ Neuroblastoma has been noted in patients with BWS whose underlying mechanism of disease includes loss of *CDKN1C* gene expression, specifically imprinting control center 2 (IC2) loss of methylation, and paternal uniparental disomy at chromosome 11p15,^51^ and has also been seen in those with intragenic variants in the maternal copy of *CDKN1C*.^52^ This clinical observation may be linked to the high embryonal expression of *CDKN1C* in the adrenal glands, from which most neuroblastomas arise.^53^ Three patients with Weaver syndrome have been reported in the literature to have neuroblastoma, and while some have hypothesized the role of insulin‐like growth factor I in the co‐occurrence of high birth weight and neuroblastoma, these hypotheses have thus far not been confirmed through laboratory sampling of patient serum levels.^54^ Additionally, while insulin‐like growth factor II (IGF2) is located in the BWS locus of chromosome 11p15, prior studies have shown that patients with BWS and tumors typically have IGF‐I and IGF‐II serum levels in the normal range.^55^ Few studies have reported *MYCN*‐amplification status in neuroblastoma cases diagnosed in individuals with congenital overgrowth syndromes, but the possible connection between this specific subtype and these syndromic conditions requires further study to guide screening and targeted therapy. Therefore, it appears reasonable that biological pathways underlying other growth phenotypes not previously explored, including height attainment, may play a role in neuroblastomagenesis given these known congenital overgrowth syndromes that confer increased risk of neuroblastoma.

Leg length, a marker of pre‐pubertal growth, is the component of stature that is most consistently associated with cancer risk.^56^ Our seemingly inconsistent result wherein increased height in childhood but decreased height in adulthood was associated with neuroblastoma risk may implicate a role for genetic determinants of pubertal timing in neuroblastoma etiology. Early onset puberty is characterized by an early growth spurt leading to taller childhood height attainment, but results in shorter overall adult height attainment.^57^, ^58^, ^59^ One limitation to our study is that data on Tanner developmental stage and age of onset of growth acceleration was not available for our case‐control sample, and since age of pubertal onset and growth acceleration can have high inter‐individual variability, we do not know whether our childhood height measurement was measured at a pre‐pubertal or post‐pubertal onset time point. However, our observation that greater childhood height attainment increased risk of *MYCN*‐amplified neuroblastoma while greater adult height attainment protected against *MYCN*‐amplified neuroblastoma suggests that that pubertal timing may play a role in in neuroblastomagenesis and merits further attention.

Interestingly, a genetic variant influencing age at menarche^13^, ^60^ was recently identified in a known *MYCN*‐amplified neuroblastoma risk gene, *LIN28B*.^61^, ^62^ This locus has previously been associated with late pubertal growth and with height attainment,^37^ providing further evidence for possible common pathways underlying pubertal timing, height attainment, and neuroblastoma etiology. *LIN28B* is often overexpressed in high‐risk neuroblastoma tumors. Suppression of *let‐7* family miRNA expression in neuroblastoma cells overexpressing *LIN28B* increases *MYCN* expression, resulting in elevated MYCN protein levels in high‐risk neuroblastomas, including those that lack *MYCN* amplification.^61^, ^62^ High expression of *LIN28B* is, therefore, a poor prognostic factor, independent of *MYCN*‐amplification status.^61^ Thus, regulation of childhood height and pubertal timing through the Lin28/let‐7 axis may influence risk of MYCN‐dependent neuroblastoma in general, not necessarily only those tumors with genomic amplification of MYCN.

Although height attainment has been well studied as a risk factor for adult‐onset cancers, the subject is significantly understudied in the context of childhood cancers. One exception is a recent publication identifying significant associations between genetic predisposition to taller childhood and adult height attainment and risk of childhood osteosarcoma.^25^ The use of polygenic score analyses has helped to resolve several challenges in the epidemiologic investigation of height attainment and childhood cancer risk. As toxic therapeutic interventions such as chemotherapy and radiation therapy can stunt growth and delay pubertal onset in neuroblastoma survivors, relationships between these anthropometric variables and neuroblastoma risk are typically confounded by the effects of treatment, which is particularly aggressive in cases of MYCN‐amplified neuroblastoma. In the present study, utilization of polygenic scores as instrumental variables for height attainment allowed us to study the association between stature and neuroblastoma risk in a case‐control setting without susceptibility to reverse causality and confounding effects due to chemo‐radiotherapy treatment.^63^, ^64^

Our study is unique in its approach to dissecting a role for genetic determinants of height in contributing to neuroblastoma risk overall and MYCN‐amplified neuroblastoma in particular. Polygenic score analyses are useful for studying the genetic epidemiology of childhood cancer, where traditional GWAS approaches are often underpowered due to sample size limitations. Such polygenic scores are calculated from variant‐effect‐size weighted aggregations of the trait‐associated alleles^64^ and can be used to encapsulate effects among a group of genetic variants that may not individually achieve significance in single SNP analyses, thereby obscuring potentially important associations.^65^, ^66^ The need to extend genetic epidemiology approaches beyond GWAS, such as through the use of polygenic scores, is increasingly essential in association studies.^67^

One limitation of our study is that we were unable to complete a formal replication of our case‐control results due to the limited incidence of neuroblastoma, which hinders the availability of independent datasets for validation; however, we sought to further validate our finding of an association between genetic determinants of height and neuroblastoma risk using rick genotype‐phenotype data available from the UK Biobank.

Our findings from UK Biobank data examining associations between height phenotypes and known neuroblastoma GWAS hits provides further validation of our case‐control finding that height attainment and neuroblastoma may share genetic drivers. Although neuroblastoma risk loci were enriched for association with both childhood and adult height phenotypes among UK Biobank participants, the neuroblastoma risk alleles were not associated with either childhood or adult stature in a consistent direction (eg, “risk alleles” associated with taller stature and “protective alleles” associated with shorter stature). Recently, we identified a similar relationship between the genetic determinants of platelet count and acute lymphoblastic leukemia (ALL) using a hybrid approach integrating case‐control and UK Biobank data.^68^ This study also did not reveal a directional relationship between genetic variants associated with platelet count and ALL risk, but rather indicated that pleiotropic genetic variants contributed to both phenotypes. Thus, while neuroblastoma risk loci are significantly enriched for association with height phenotypes compared to our control SNP sets, these associations do not suggest that stature itself is likely to act as an effect mediator (ie, “intermediate phenotype”)^63^ on a causal pathway connecting these genetic variants to neuroblastoma pathogenesis. Instead, it is likely that there are common biological pathways influencing both height attainment and neuroblastomagenesis, although our analyses did not identify specific pathways (eg, WNT or Hedgehog) that seemed to drive the polygenic score associations.

Our observations that genetic determinants of both taller pre‐pubertal height and shorter adult height were independently associated with risk of MYCN‐amplified neuroblastoma reinforce the idea that distinct genetic risk factors and biological pathways underlie risk of MYCN‐amplified vs unamplified neuroblastoma. These findings reveal that there are likely pleotropic genetic determinants that influence both height/pubertal timing and neuroblastomagenesis. These shared biological pathways and the genetic architecture underlying both growth trajectories and risk of MYCN‐amplified neuroblastoma require additional investigation. Further exploration of this novel association may potentially improve prognostication and risk stratification of children with neuroblastoma by leveraging germline genetic risk variants and may reveal therapeutic targets for aggressive MYCN‐amplified neuroblastoma.

## CONFLICT OF INTEREST

The authors declare no conflicts of interest.

## ETHICS APPROVAL

The study was approved by the Institutional Review Boards at Duke University (Pro00087859) and dbGaP data were accessed after approval by the NCI data access committee for the study “Post‐GWAS discovery of cancer predisposition genes in pan‐cancer datasets”.

Datasets used for the analyses described in this manuscript were obtained from dbGaP study accession phs000124.v2.p1 [Neuroblastoma Genome‐Wide Association Study (NBL‐GWAS)]. The Neuroblastoma genome‐wide association study is made possible through a collaboration between the Children's Hospital of Philadelphia (CHOP), the University of Pennsylvania and the National Cancer Institute‐funded Children's Oncology Group (COG; U10‐CA98543). Cases were identified through the COG Neuroblastoma Tumor Bank, and controls recruited at CHOP. This work was funded by NIH grant R01‐CA124709, the Giulio D’Angio Endowed Chair at CHOP, the CHOP Research Endowment, the Alex's Lemonade Stand Foundation, the Evan Dunbar Foundation, the Rally Foundation, Andrew's Army Foundation and the Abramson Family Cancer Research Institute. Additional controls were accessed from Illumina's iControlDB database.

## Supporting information

Supplementary Material

## Data Availability

Datasets used for the analyses described in this manuscript are available from dbGaP –Neuroblastoma Genome‐Wide Association Study (study accession phs000124.v2.p1).
